# To Dr. Bradley

**Published:** 1805-11-01

**Authors:** W. Patterson


### To Dr. BRADLEY.

SIR,

THE Reports which certain public associations have circulated, that the vaccine inoculation has not been embraced in Ireland with the zeal which its character merits, is too general a charge; for, in some districts, even country gentlemen inoculated, with presumed success, numbers of children who were brought in groups to their country seats for that purpose; and, in some parts of Ireland, the practice was early adopted by medical practitioners. I adopted it, in April 1801, and was the first person who introduced it into the north-west district of Ulster. Of my practice, particularly with respect to atmospherical influence, I intend to publish an account. If it have not spread in Ireland, with the rapidity it has done in other countries, we may in future have reason to congratulate ourselves on the slowness of its progress, whence, according to the proverb, may result its sureness; for I am apprehensive, that the enthusiastic rapidity with which it is pursued in some countries, where thousands and tens of thousands are inoculated with a sort of magical slight, will eventually injure its repute. Here, (Derry), where it is gaining ground, the small-pox was not, in my memory, (thirty-three years back), so long absent as it has been since vaccination began to increase, which has been the case for more than a year past.

I am, &c.

W. PATTERSON.

*October* 8, 1805.

